# The value of routine histopathological examination of appendicectomy specimens

**DOI:** 10.1186/1471-2482-7-17

**Published:** 2007-08-10

**Authors:** Alun E Jones, Alexander W Phillips, John R Jarvis, Kevin Sargen

**Affiliations:** 1 Department of General Surgery, Norfolk and Norwich University Hospital NHS Trust, Colney Road, Norwich, NR5 7UR, England, UK

## Abstract

**Background:**

Appendicectomy specimens removed from patients with suspected acute appendicitis often appear macroscopically normal but histopathological analysis of these cases may reveal a more sinister underlying pathology. We evaluated histopathological reports of 1225 appendicectomy specimens at the Norfolk and Norwich University Hospital (NNUH) over the past three years.

**Methods:**

Histopathology reports for all appendices analysed at the NNUH between March 2003 and March 2006 were reviewed by examination of the case notes. The analysis focussed on the confirmation of acute appendicitis, incidental unexpected incidental findings other than inflammation, whether these abnormalities were suspected on gross examination at the time of surgery, and the effect on patient management and prognosis.

**Results:**

The histopathology reports disclosed a variety of abnormal incidental lesions. Of the 1225 specimens, 46 (3.75%) revealed abnormal diagnoses other than inflammatory changes. Twenty-four (1.96%) of these were clinically significant and affected further patient management. Only two of these (0.16%) were suspected on macroscopic examination intra-operatively.

**Conclusion:**

Twenty-four of the 1225 specimens (1.96%) had an impact on patient management or outcome and were not suspected on macroscopic examination at the time of surgery. These would have been missed had the specimens not been examined microscopically. The intra-operative diagnosis of the surgeon is therefore unreliable in detecting abnormalities of the appendix. This study supports the sending of all appendicectomy specimens for routine histopathological examination.

## Background

Acute appendicitis is the most common general surgical emergency [1]. In England 42,526 patients underwent appendicectomies in the year 2004–5 with a mean age of 28 years [2]. Approximately 20% of those undergoing appendicectomes are found not to have acute appendicitis at surgery [3-6], with this being more common in females than males and approaching a ratio of 3:1 in the 15–19 age group [7].

Clinical findings form the basis for diagnosis which may be consolidated by blood tests such as C-reactive protein and white cell count, and management is early appendicectomy [8]. The practice of sending appendicectomy specimens for histopathological analysis varies [9]. Whereas it is recognised that many resected specimens in general surgery need not be sent, there are as yet no guidelines as to whether all appendices should be sent as a matter of routine [10]. Matthyssens *et al *suggest that appendices should not routinely be sent unless there is an obvious macroscopic abnormality at surgery [9]. They argue that this practice is justified by the rarity of aberrant findings, together with the significant costs of specimen processing, an ever increasing issue in the current financial climate. However, a number of previous papers have found aberrant incidental findings to be more common, suggesting that this latter method has the potential to miss significant pathologies which may impact on patient management [11-16].

Less than 50% of appendiceal tumours are identified intraoperatively [17]. Further, parasitic infections, endometriosis, inflammatory bowel disease may be picked up from appendix specimens. There is also evidence that "normal" appendices may have evidence of an inflammatory pathologic condition, which is only obvious at a molecular level [13].

We report on the incidence of unexpected pathology in resected appendix specimens at the Norfolk and Norwich University Hospital.

## Methods

Between March 2003 and March 2006, 1225 patients underwent appendicectomy for a clinical presentation consistent with acute appendicitis. There were 642 males (52%) and 583 females (48%), with a mean age of 32 years (range 6 months to 90 years). Three-hundred and thirty-nine were removed laparoscopically (28%), 886 (72%) open, with an increasing trend towards laparoscopic removal over the past twelve months.

Operative records stored electronically on the ORSOS (Operating Room Scheduling Office System) database were searched for "appendicectomy, emergency" from March 2003 to March 2006 inclusive. The histopathology reports of this cohort of patients were examined by searching the case notes. Findings were recorded as (a) evidence of acute appendicitis (including perforation and gangrene) and (b) incidental, abnormal findings. The case notes of the abnormal cases were further examined for subsequent investigations, follow-up and outcomes.

Appendicectomy specimens are prepared according to a hospital-defined protocol, involving immediate fixing in formalin prior to transport to the pathology laboratory. Specimens are sectioned at the tip, body and base and are examined by a consultant or senior pathologist. Details of macroscopic and microscopic findings are issued in the final report.

The pathology request forms submitted with the specimen and operative notes of the unexpected abnormal cases were reviewed for evidence that the pathologies were suspected on gross examination of the appendix by the surgeon intra-operatively.

The patient notes of abnormal findings were also reviewed to determine the clinical significance of the findings. A result was defined as being clinically significant if further follow up investigations (such as staging CT, colonoscopy, biopsy) or further surgical management was required, or if the result affected patient prognosis.

## Results

A total of 1225 reports were examined. Of these, 941 (77%) reported changes consistent with acute inflammation (acute appendicitis, abscess and perforated gangrenous appendicitis). Two-hundred and eighty four (23%) appendices were within normal limits. Forty-six specimens revealed incidental abnormal diagnoses. Twenty-four of these were clinically significant and two were suspected by the operating surgeon (carcinoids). Eleven of these revealed intraluminal parasites (10 *Enterobius *and 1 *Schistosoma*), 3 showed endometriosis and 6 showed Crohn's disease. Twenty-three showed benign tumours or tumour-like conditions (13 carcinoids, 6 mucinous cystadenomas and 4 hyperplastic polyps). Three cases of malignant tumours were identified, 2 of which were primary adenocarcinomas and one of which was a metastatic ovarian adenocarcinoma. (See Table 1).

**Table 1:** Numbers of unexpected findings from appendicectomy specimens

	**Number of Cases**		
**Diagnosis**	**Macroscopic**	**Microscopic**	**Total**
**Parasites**			
*Enterobius*	0	10(10)	10(10)
*Schistosoma*	0	1(1)	1(1)
**Endometriosis**	0	3	3
**Crohn's disease**	0	6(3)	6(3)
**Benign Tumours**			
Hyperplastic polyp	0	4	4
Cystadenoma	0	6(2)	6(2)
Carcinoid	2(2)	11(3)	13(5)
**Malignancy**			
Primary adenocarcinoma	0	2(2)	2(2)
Secondary adenocarcinoma	0	1(1)	1(1)
**Total**	**2(2)**	**44(22)**	**46(24)**

Figures in brackets are those deemed "clinically significant".

### Incidental abnormal appendices/Treatment/Patients' Outcome

All 11 cases of parasitic infestation were treated with antihelmithic drugs, in many cases by contacting the GP following the issue of the report.

All hyperplastic polyps were less than 5 mm in diameter, confined to the appendix tip, showed no evidence of dysplasia and were fully resected with the specimen. No further follow up was arranged.

All suspected cases of Crohn's disease underwent barium follow through and colonoscopy with biopsies. In 3 of these cases, active Crohn's disease was confirmed and the patients referred to the gastroenterologists.

All the mucinous cystadenomas were histopathological diagnoses. Three were suspected on gross examination by the pathologist and confirmed on sectioning. The remaining 3 showed the characteristic mucin-filled lumen on sectioning. Four mucinous cystadenomas were benign lesions and were fully resected. However, 2 showed severe dysplastic change and were later discussed at the multi-disciplinary team (MDT) meeting. One patient went on to have colonoscopy and caecal biopsies, which were negative for tumour. The other underwent CT abdomen and pelvis, which revealed no abnormalities. Both patients are currently being monitored as out-patients with regular colonoscopies in line with the colon cancer protocol.

Of the 13 carcinoids, 8 were completely resected. Five were not completely resected and required follow-up. All 5 were clinically significant. Only 2 of these were suspected by the surgeon intra-operatively on the basis of finding a firm yellow material at the tip. Both were large tumours (2.5 cm and 1.6 cm) with focal invasion of the mesoappendix. Although the resection margins were tumour-free in both cases, their large diameter was predictive of a high risk of recurrence and metastasis. Following MDT discussion, right hemicolectomy was performed on one of these cases. The other was also discussed at MDT and further resection deemed unnecessary due to the negligible mitotic rate. This patient as had follow-up CT scans due to unrelated renal pathology, which have revealed no further problems with respect to the bowel.

Of the remaining three carcinoids, all were less than 1 cm and diagnosed microscopically and all underwent right hemicolectomy.

The 3 cases of adenocarcinoma were all microscopic findings. Two were primary tumours and underwent right hemicolectomy. The pathologist's report subsequently confirmed complete clearance. The third was a metastatic ovarian carcinoma and received palliative care.

## Discussion

The histopathological examination of the appendix serves two purposes. First, it allows the diagnosis of acute appendicitis to be confirmed, especially where this is not evident intra-operatively. Second, histopathological examination may disclose additional pathologies that may not be evident on gross examination intra-operatively but may affect subsequent clinical management of the patient. Specimens reported as negative for acute appendicitis are useful in eliminating acute appendicitis as a cause of symptoms and allowing further investigations to be performed should symptoms persist. Even in these negative appendicitis cases, patients' symptoms frequently disappear post-operatively. It has been suggested that in these cases there may be an early sub-clinical appendicitis [13].

The examination of certain surgical specimens, such as hernia sacs, and "doughnuts" following rectal surgery, does not yield further useful information and is unnecessary [9,10]. However, there are very few studies, which evaluate the benefits of analysing appendicectomy specimens. As a result, some centres, including this one, send all resected appendices for histopathological analysis. Other centres send specimens only if they appear macroscopically abnormal at the time of surgery [9]. This latter practice has the potential to miss important diagnoses which may subsequently affect patient management and is illustrated in our study, where evaluation of the histopathology reports of 1225 specimens revealed 46 unexpected findings of which 24 were clinically significant. Other authors have similar experiences. Polat *et al *report an intra-operative detection rate of less than 50% for all types of appendiceal tumour [12]. Deans *et al*, suggested that surgeons missed abnormal pathological findings in 10 out of 13 patients, the majority of which required further investigation or treatment [15].

In this study histopathological examination disclosed a variety of lesions. *Enterobius *infection is often associated with acute appendicitis by causing intraluminal obstruction, and may be effectively eradicated by anti-helminthic treatment [11]. Hyperplastic polyps have very little malignant potential and have no known important clinical associations [11]. Mucinous cystadenomas are premalignant and may be associated with synchronous large bowel lesions [11]. Carcinoid tumours were diagnosed in 13 (1.05%) specimens, an incidence more than three times higher than quoted in other studies [9,19]. Adenocarcinoma accounted for 3 (0.24%) cases, consistent with figures from other studies [19]. Most benign tumours are cured by appendicectomy alone [14]. However, there are a number of cases in which right hemicolectomy is indicated [14]. These include all adenocarcinomas, tumours invading the mesappendix, serosa, lymphatics or vasculature, and benign tumours with a diameter greater than 2 cm. Similarly, right hemicolectomy is preferred for tumours of greater than 2 cm on CT. Benign tumours of diameter 1–2 cm may be treated according to the surgeon's discretion [15]. For CT appearances of up to 1 cm appendicectomy is adequate [15]. All patients with appendiceal tumours should be followed up because a secondary malignancy may develop in up to 20% of them [15].

Table 1 illustrates the variety of incidental findings disclosed by the pathologist. Twenty-four out of these 46 incidental abnormal diagnoses had a significant impact on patient management. Only 2 were suspected on gross examination. Twenty-two were not identified on gross examination and would have been missed had the specimen not been sent for routine histopathological analysis.

## Conclusion

This study illustrates that intra-operative detection of abnormal appendices by the surgeon is unreliable and supports the sending of all appendicectomy specimens for routine histopathological analysis.

## Competing interests

The author(s) declare that they have no competing interests.

## Authors' contributions

AEJ and AWP conceived the study, collected and analysed the data and drafted and referenced the article. JRJ and KS reviewed the draft and made corrections and suggestions for improvement. AEJ revised the article. All authors read and approved the final draft.

## Aknowledgements

The study was funded by the authors. There are no additional acknowledgements.

## Pre-publication history

The pre-publication history for this paper can be accessed here:


